# Beyond Stereoisomeric Effects: Exploring the Importance of Intermolecular Electron Spin Interactions in Biorecognition

**DOI:** 10.1021/acs.jpclett.3c01595

**Published:** 2023-07-31

**Authors:** Yiyang Lu, Meera Joy, Brian P. Bloom, David H. Waldeck

**Affiliations:** † Chemistry Department, University of Pittsburgh, Pittsburgh, Pennsylvania 15260, United States

## Abstract

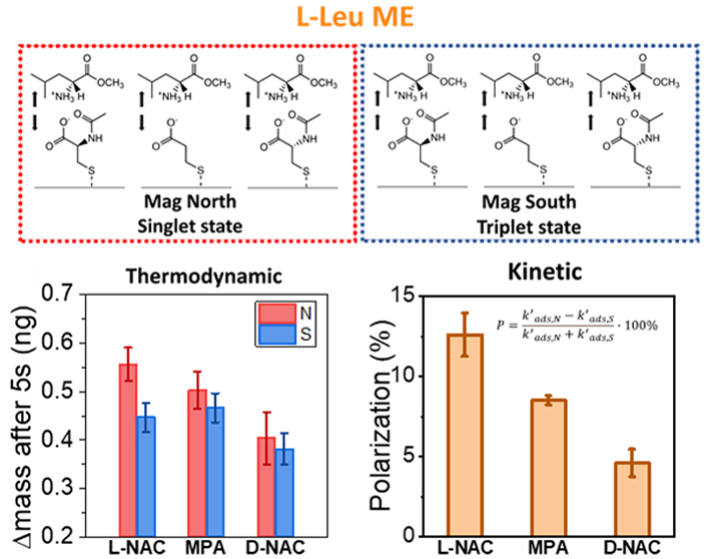

This work shows that electron spin polarization and stereoisomeric effects make comparable contributions to the enantioselective binding of amino acids. Magneto-electrochemical quartz crystal microbalance methods are used to study the adsorption of chiral amino acids onto a monolayer film of chiral molecules that are spin polarized by an underlying ferromagnetic substrate. The direction of the electron spin polarization affects both the kinetics and thermodynamics of the enantiospecific adsorption of the amino acids. Comparison of these data with the circular dichroism (CD) spectra of the amino acid adsorbates shows that the CD spectrum of the interacting group provides a good figure-of-merit for predicting the contributions of electron spin to the intermolecular interaction. These findings demonstrate the importance of electron spin in enantioselective intermolecular interactions between chiral amino acids and represent a paradigm shift for how selectivity should be viewed in biorecognition.

Chiral molecules comprise the fundamental building blocks of life and appear essentially in an enantiopure form (homochiral); e.g., sugars are primarily found in the d-configuration, while natural amino acids predominantly occur in the l-configuration.^1,2^ Although the emergence of homochirality in nature remains an enigma, it is well-known that enantiomeric purity has important consequences for biochemical processes;^3,4^ e.g., despite possessing the same bond connectivity, the “wrong” enantiomer can have detrimental effects.^5^ Such behavior is particularly important in pharmacological applications, and ongoing research efforts aim to improve enantioselective synthesis^6−8^ and chiral resolution strategies.^9−11^ Conventional wisdom maintains that enantioselectivity in molecular recognition by biomolecules arises from differences in the three-dimensional binding geometries and their charge distributions;^12−14^ however these considerations do not aqccount for the role that the molecules’ electron spin polarization may play in these processes.

Research over the past 20 years shows that chiral molecules can spin polarize electrons upon transport and charge redistribution, owing to the chiral induced spin selectivity (CISS) effect,^15−17^ and that CISS-mediated processes occur in biological systems.^17−20^ The CISS effect refers to the phenomenon in which electrons with a defined spin orientation preferentially transmit through chiral molecules and materials, whereas transport of electrons with the opposite spin orientation is inhibited. Note, the preferred spin transport depends sensitively on the enantiomeric form of the molecule, or enantiomorph of the material, through which it traverses.^21^ Because the charge polarization of a chiral molecule is accompanied by a net spin polarization,^22^ we posit that electron spin polarizations ought to manifest in biomolecular intermolecular interactions.

This work uses magneto-electrochemical quartz crystal microbalance (mEQCM) methods to study the adsorption kinetics of amino acids onto magnetized ferromagnetic surfaces, which are coated with *N*-acetyl cysteine (NAC) self-assembled monolayer (SAM) films of chiral molecules. Note that NAC is a common mucolytic agent and is used as a pharmaceutical for treating various biological disorders in addition to sustaining the production of glutathione *in vivo*; see ref (23) for a comprehensive review of the role of NAC and its interactions at the organ, tissue, and cellular levels. Here, amino acids are used to represent common biological adsorbates, and the NAC SAM/ferromagnetic electrode is an analogue for a biomacromolecule possessing spin polarization. We show that the intermolecular binding is enantio- and spin-specific and that chiroptical features of the adsorbate are a good predictor for determining the sign of the spin selectivity. It is important to stress that the spin dependence on intermolecular interactions ought to exist among all types of chiral molecule–chiral molecule interactions and thus is an essential feature for directing biological processes. More broadly, these findings elucidate new features of the mechanism underlying spin-driven enantiospecific crystallization from racemic mixtures^24^ as well as enhance our understanding of enantiospecific adsorption of chiral molecules onto magnetized ferromagnetic surfaces.^25^

To probe the spin-dependence in the binding of amino acids, we prepared self-assembled films of an amino acid analogue on a ferromagnetic surface and studied the binding of amino acids to that film as a function of its magnetization state, following similar methods to that established in our previous work.^25^ Briefly, an external magnetic field was applied to the underside of the electrode with its North or South pole oriented normal to the electrode surface, and chronoamperometric techniques were used to monitor the change in mass and hence the kinetics for the adsorption; see Figure 1a for a schematic illustration of the setup and the Supporting Information for additional experimental details. Here, the adsorption kinetics of leucine methyl ester, LeuME, onto *N*-acetyl cysteine, NAC, self-assembled monolayer (SAM) coated Ni/Au electrodes (100 nm Ni/10 nm Au/NAC) electrodes was studied. An applied bias potential of −0.4 V was used to facilitate adsorption of LeuME onto the electrode and then the potential was jumped to 0 V to initiate desorption. Note, Supplementary Figure S1 shows control experiments which demonstrate that under these potential conditions the NAC remains on the electrode surface and only adsorption/desorption of LeuME occurs.

**Figure 1:**
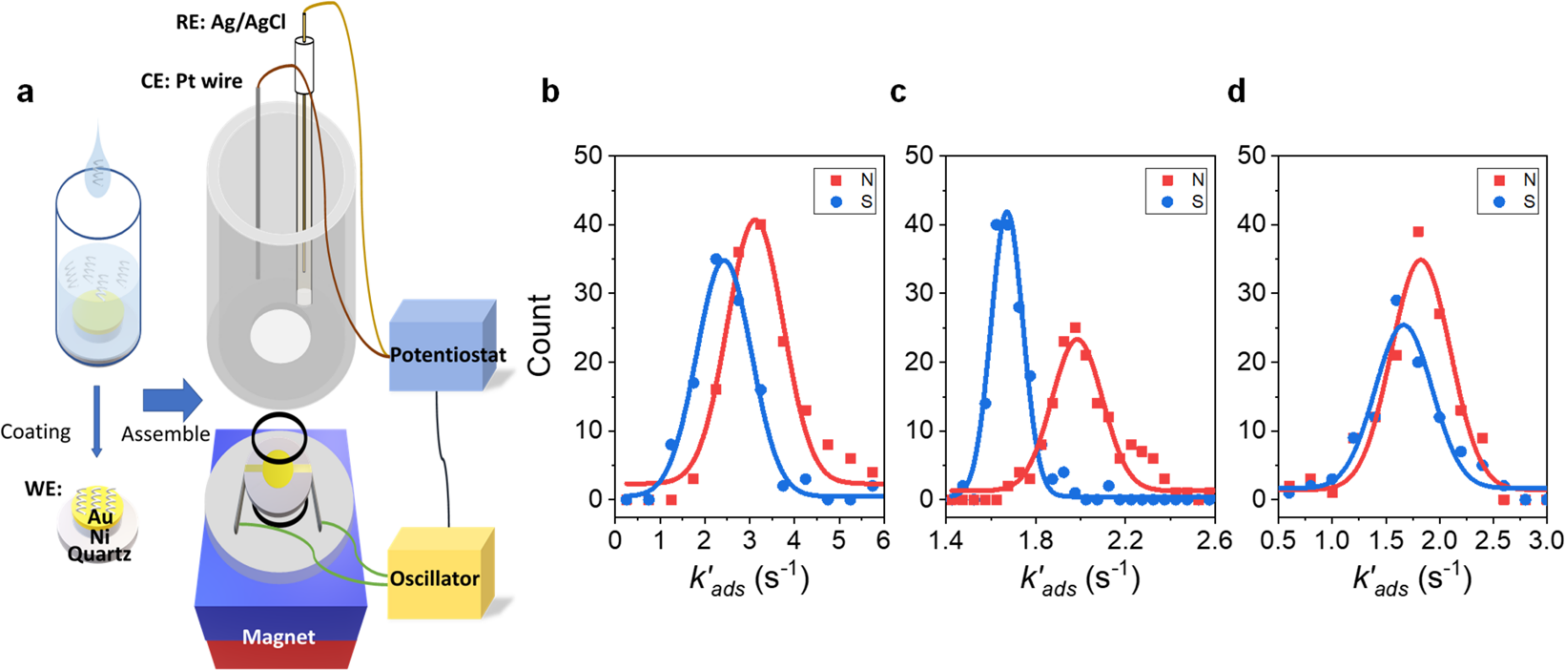
Determination of adsorption rate constants. (a) Schematic illustration of the mEQCM set up. (b–d) Histograms for the adsorption rate constant of l-leucine methyl ester (l-LeuME) onto Ni/Au film electrodes coated with l-N-acetyl cysteine (l-NAC), mercaptopropionic acid (MPA), and d-NAC monolayer films, respectively, under North magnetic field (red) and South magnetic field (blue). Note, the solid line is a Gaussian fit to the data.

Figure 1 shows histograms of the measured adsorption rate constants of l-LeuME onto l-NAC (Figure 1b), mercaptopropionic acid, MPA, as an achiral control (Figure 1c), and d-NAC (Figure 1d) coated electrodes for the cases of a North magnetized electrode (red) and a South magnetized electrode (blue). For all three SAM compositions, the adsorption rate constant for l-LeuME is faster when the magnetization is oriented North rather than South. Conversely, the opposite is true, as South is faster than North, when d-LeuME is used as the adsorbate; see Supplementary Figure S5. The dissymmetry in the magnetization dependence of the adsorption rate constant with the amino acid’s enantiomeric form is consistent with a CISS-mediated effect.^26^ To quantify the magnitude of the dissymmetry in adsorption rate constant with magnetization, a polarization parameter *P* was defined as eq 1
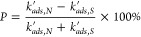
1where *k*^′^_*ads*,*N*_ and *k*^′^_*ads*,*S*_ correspond to the average adsorption rate constant, determined by the maximum of a Gaussian fit to the histogram data, under North and South magnetizations of the electrode, respectively.

Figure 2 shows *P* values for l-LeuME (orange) and d-LeuME (green) on l-NAC, d-NAC, and MPA SAM-coated Ni/Au electrodes and l-NAC coated Au electrodes as a control experiment. For these data, the sign of *P* is determined by the chirality of the adsorbate; however, the magnitude of *P* changes with the enantiomeric form of the SAM relative to the adsorbate. *P* for the achiral MPA SAM has nearly equal magnitude for the two different LeuMe adsorbates, albeit with opposite signs. These data reveal the effect of spin polarization, which is driven by the magnetized film electrode, on the binding to MPA, which should not have a stereoisomeric preference for one amino acid enantiomer over the other and should not display spin polarization arising from the charge polarization because it is achiral. On the other hand, the l-NAC films show a larger magnitude of *P* for the binding of l-LeuME than it does for the MPA or for the d-LeuME. Correspondingly the homochiral binding of d-NAC with d-LeuME has a larger magnitude of *P* than its heterochiral analogue of d-NAC with MPA and l-LeuME but with different signs. While the difference in the sign of *P* is controlled by the spin polarization preference of the analyte amino acid, the magnitude of *P* is affected by the spin polarization created in the SAM by the magnetized electrode,^19^ the spin-induced charge redistribution in the chiral SAMs,^27^ and the stereoisomeric interactions between the amino acid and the chiral SAM.^28^

**Figure 2:**
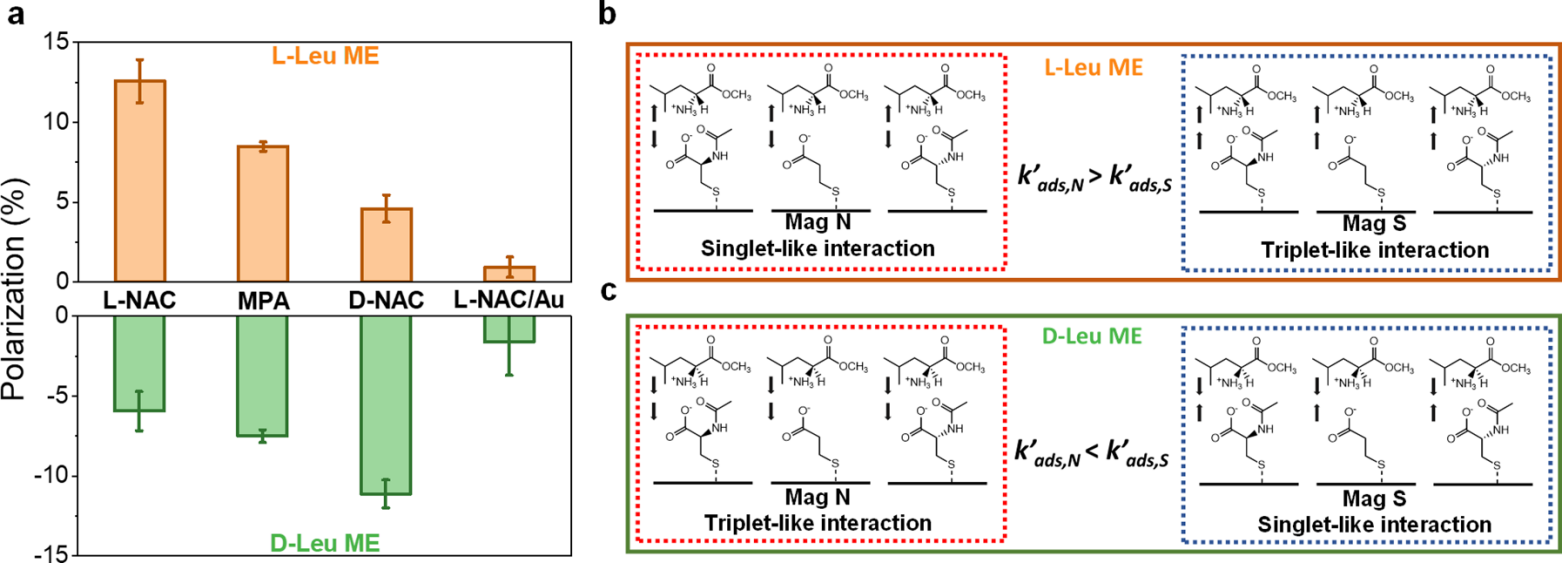
Spin mediated asymmetry in adsorption kinetics. (a) Polarization in adsorption rate constant for l-LeuME (orange) and d-LeuME (green) enantiomers on three different monolayer films: l-NAC, MPA, and d-NAC on Ni/Au substrates, and for l-NAC coated on an Au substrate as a control experiment. The error bars represent the uncertainty and were obtained by propagating the error from eq 1, using the standard deviation of the mean of the rate constant as an error measure. (b) Mechanistic scheme illustrating the emergence of an intermolecular “singlet-like” adsorption interaction between l-LeuME with different SAMs under a North magnetic field and a “triplet-like” adsorption interaction under a South magnetic field. (c) Mechanistic scheme illustrating the emergence of an intermolecular “triplet-like” adsorption interaction between l-LeuME with different SAMs under a North magnetic field and a “singlet-like” adsorption interaction under a South magnetic field. See text for a more detailed discussion of the mechanism.

The scheme in Figure 2 shows a proposed mechanism for the spin- and enantiospecific adsorption of l-LeuME onto the three SAM electrodes. As l-LeuME approaches the negatively charged l-NAC SAM-coated surface, charge redistribution of the l-LeuME molecule’s electron cloud occurs. Because of the CISS effect, the induced charge polarization gives rise to an accompanying spin polarization across the molecule, with spin up electrons accumulating at the −NH_3_^+^ terminus. As the l-LeuME nears the SAM, electron exchange interactions increase between the spin polarized −COO^–^ in l-NAC, determined by the magnetization of the substrate, and the spin polarized −NH_3_^+^ in l-LeuME, determined by the handedness of the molecule. The attractive interaction is stronger when the excess spin density at the surface of the SAM is opposite to that of the excess spin density presented by the molecule in solution, i.e., a “singlet-like” interaction, than it is for the case where the spin density of the SAM and the adsorbate molecule are parallel, i.e. a “triplet-like” interaction.^19,22^ Thus, the l-LeuME, in which the −NH_3_^+^ terminus adopts an “up” spin, interacts with the North magnetized surface, net spin down, with a singlet-like interaction (pairing) and displays faster adsorption kinetics; see Figure 2b, left. Conversely, when the surface of the electrode is South magnetized, the excess spin density at the surface possesses the same orientation as that on the l-LeuME −NH_3_^+^ terminus, “up” spins, giving rise to a “triplet-like” interaction and slower adsorption kinetics; see Figure 2b, right. Note that the use of “singlet-like” and “triplet-like” refer to the spin–spin interaction alignment and not specifically to singlet and triplet electronic states. Because the excess spin density at the surface of the electrode is always defined by the applied magnetic field direction, the l-LeuME always adsorbs faster with North magnetization than South magnetization; the opposite is true for d-LeuME (see Figure 2c). Control experiments on an l-NAC coated Au diamagnetic electrode show only weak differences in the adsorption rate with magnetization; *i.e.*, *k*_*ads*,*N*_^′^ and *k*_*ads*,*S*_^′^ of l-LeuME (or d-LeuME) are nearly equal. Altogether these data reveal the pronounced effect that spin can have on the adsorption kinetics.

The magnitude of the excess spin polarization, and hence the magnitude of *P*, depends on the amount of spin density emanating from the ferromagnetic electrode through the SAM. Previous work on chiral SAM-coated ferromagnetic electrodes shows that changing the electrode’s magnetization, North versus South, generates a change in the surface charge that also depends on the handedness of the SAM; i.e., spin polarization elicits a change in charge polarization.^27^ For our system, l-NAC (d-NAC) SAMs facilitate spin “down” (“up”) electrons more favorably than spin “up” (“down”) electrons which implies that the surface charge and double layer potential drop is likely larger under a North (South) magnetic field. This magnetization-dependent change in surface charge should accompany the standard stereoisomeric differences that manifest for homochiral/heterochiral effects on the intermolecular interaction energies.

To test this hypothesis, we compared the change in mass of l-LeuME at 5 s, after the adsorption process has largely concluded. Figure 3 reports these data for l-NAC, MPA, and d-NAC coated electrodes as a function of magnetization. For the different SAM compositions, a difference in adsorbate mass was observed under North and South applied magnetic fields, and the average mass, (*m*_north_ + *m*_south_)/2, adsorbed on the three SAMs is different; see Supplementary Table S1. Homochiral ensembles exhibited the largest average change in mass and the largest mass asymmetry with magnetic field, whereas the heterochiral assemblies exhibited the smallest average mass and the smallest mass asymmetry with magnetic field. The achiral SAMs were intermediate between the homochiral and heterochiral cases. Previously, the enantiospecific adsorption of chiral molecules onto a bare ferromagnetic substrate was determined to be a kinetically controlled process.^25,29^ For the biomimetic system used here, the experiments reveal both kinetic and thermodynamic contributions to the enantiospecific binding, suggesting a synergy between the spin polarization emanating from the ferromagnetic electrode and the CISS response of the chiral NAC film.

**Figure 3:**
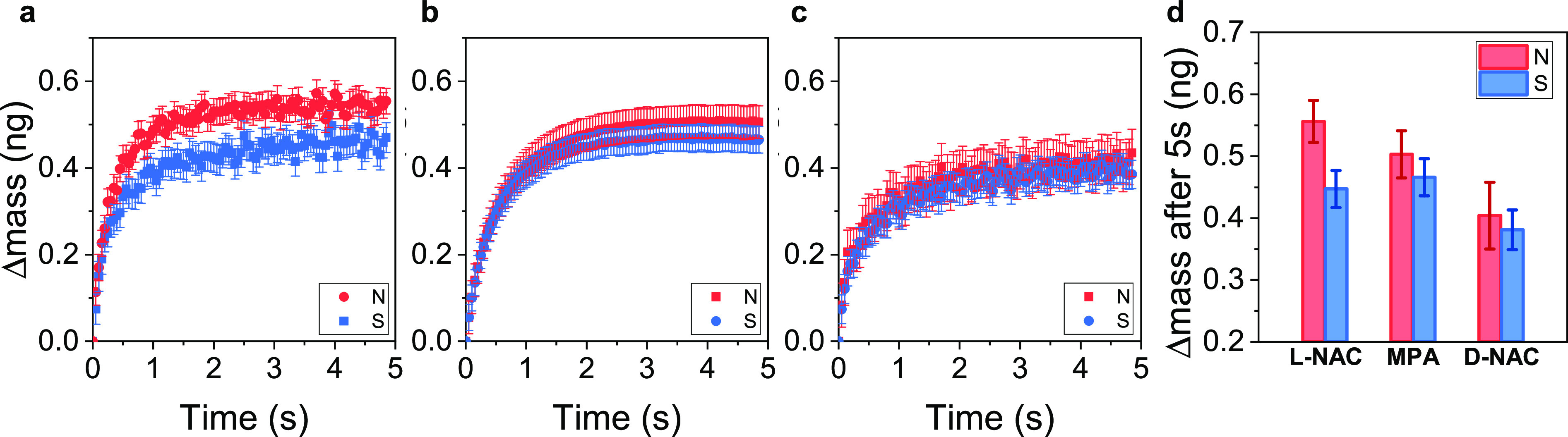
Magnetic field effects on amount of adsorption. (a–c) Average mass change during the adsorption of l-LeuME onto Ni/Au substrate coated with l-NAC, MPA, and d-NAC, respectively, under North magnetic field (red) and South magnetic field (blue). The error bars represent the standard deviation of the mean of the mass across multiple measurements. (d) Average mass change at 5s on different monolayer films: l-NAC, MPA, and d-NAC on Ni/Au substrate. The error bars represent the standard deviation of the mean of the mass.

Figure 4a illustrates the stereoisomeric effect on the intermolecular interaction, where more molecules can adsorb for homochiral ensembles compared with heterochiral ensembles. Figure 4b illustrates the combination of stereoisomeric and spin effects. The largest mass change, strongest intermolecular adsorption, occurs for homochiral ensembles under a certain magnetic field direction; i.e., l-LeuME with l-NAC SAMs under a North magnetic field. Magnetization of the substrate affects the surface charge, and this effect operates in concert with the stereoisomeric effects on the binding. These effects act over long times and can give rise to the total mass change that is observed. The chiral films also display a spin polarization, giving rise to singlet-like vs triplet-like interactions between the l-LeuME and the l-NAC layer, and these may operate primarily on the rate of adsorption. Thus, the combined system of chiral SAMs and ferromagnetic substrates can display both kinetic and thermodynamic differences; i.e., North magnetization (Figure 4b(i)) of the Ni/Au/l-NAC interfaces gives rise to higher adsorption rate constants and to higher amounts of adsorbed l-LeuME than South magnetization does (Figure 4b(ii)). For heterochiral assemblies, the intermolecular interaction energies should be weaker. For Ni/Au/d-NAC interfaces the North magnetized electrode produces a lower excess spin density in the “down” direction than it does for the l-NAC case, and this leads to a smaller shift in the interfacial charge. These effects combine with stereoisomeric differences to reduce the overall spin-dependent adsorption as compared to the homochiral case.

**Figure 4:**
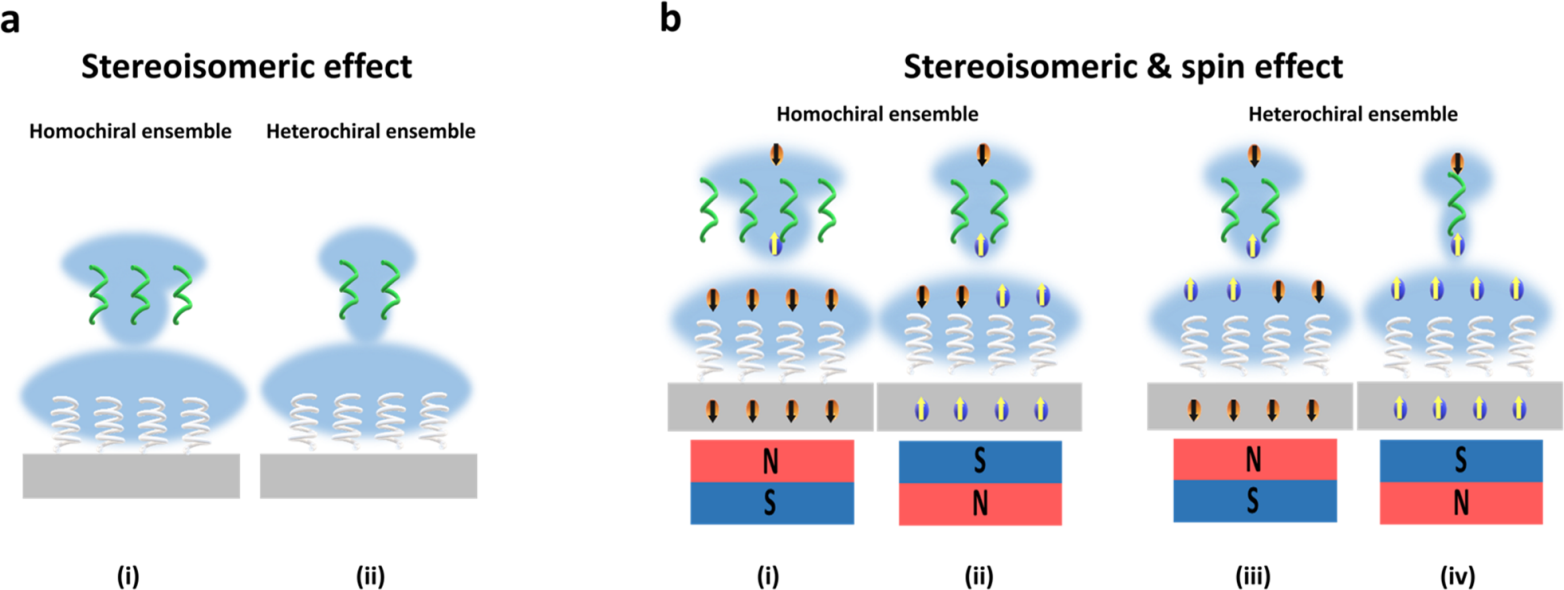
Stereoisomeric and spin effect contributions to adsorption. (a) Schematic representation of stereoisomeric effect for the intermolecular interaction for homochiral ensemble and heterochiral ensemble. (b) Schematic representation of stereoisomeric and spin effects for the intermolecular interaction of homochiral ensembles and heterochiral ensembles under North and South magnetic fields. The size of the electron cloud, blue, represents the magnitude of the charge density. Spin down electrons are depicted as orange and spin up electrons depicted as blue.

To assess the universality of the findings for the LeuME to NAC SAM system, additional experiments were performed using l-NAC as the SAM with other levorotatory amino acids. The polarizations from these studies are reported in Table 1. Experiments showed that l-phenylalanine, l-tryptophan, l-histidine, l-proline, and l-tyrosine amino acids exhibit negative polarizations in the adsorption rate constants, whereas l-leucine, l-isoleucine, l-serine, l-alanine, and l-leucine methyl ester amino acids display positive polarizations. Table 1 also reports findings for the achiral amino acid glycine, for which no net polarization is observed.

**Table 1:** Polarization in Adsorption Rate Constant of l-Amino Acids onto l-NAC SAMs and the Peak Sign of the CD Spectra of l-Amino Acids in the 190–200 nm band^a^

	Polarization	Sign of CD at 190–200 nm
l-Phenylalanine (l-Phe)	–12.64 ± 0.94%	–
l-tryptophan (l-Trp)	–7.56 ± 0.17%	–
l-Histidine (l-His)	–12.6 ± 1.75%	–
l-Leucine (l-Leu)	2.97 ± 0.28%	+
l-Isoleucine (l-Ile)	2.68 ± 1.10%	+
l-Serine (l-Ser)	3.80 ± 0.62%	+
l-Alanine (l-Ala)	1.90 ± 0.39%	+
l-Proline (l-Pro)	–5.93 ± 0.51%	–
l-Tyrosine (l-Tyr)	–6.90 ± 0.36%	–
l-Leucine Methyl Ester (l-LeuME)	12.59 ± 1.35%	+
Glycine (Gly)	0.88 ± 0.58%	N/A

a The error in polarization is the uncertainty calculated from eq 1 with the standard deviation of the mean of the rate.

Because earlier work on spin-dependent electron transfer rates and spin-filtering of electron currents by chiral molecules has shown a correlation of the measured spin polarization with the system’s chiroptical response,^30^ the polarization data for the adsorbate binding were compared with the circular dichroism (CD) spectra for each amino acid (see Supplementary Figure S13). Table 1 reports the sign of the CD signal between 190 and 200 nm, with a majority contribution corresponding to a high intensity π–π* transition associated with the π orbitals of the carbonyl bond as well as contributions from the amine.^31,32^ Interestingly, for amino acids displaying a negative *P* in the adsorbate kinetics, the CD signals between 190 and 200 nm are negative; whereas for amino acids showing a positive *P* in the adsorbate kinetics, the CD signals between 190 and 200 nm are positive. It is important to appreciate that different features in the CD spectra can emerge for the different amino acids (associated with constraints of the molecular backbone,^33^ aggregation through hydrophobic aromatic side chains,^31^ and peak shifts arising from electronic effects^31,34^); however, the correlation of the sign of the *P* with the sign of the CD signal associated with the interacting moieties persists.

This work shows that the intermolecular interactions between a biomolecular analogue and chiral molecules are enantiospecific and strongly sensitive to electron spin effects. The spin-dependent exchange interactions manifest as changes in both the kinetically driven adsorption process, i.e., singlet-like vs triplet-like interactions control the adsorption rate constant, as well the thermodynamically controlled surface energies, leading to differences in the amount of adsorbate on the different spin polarized surfaces. The spin-dependent phenomena are shown to occur in conjunction with traditional changes in intermolecular interaction energies associated with homo and heterochiral architectures. The findings show that the spin polarization effects are comparable, even stronger, than the stereoisomeric effects. Lastly, we demonstrate that the sign of the CD signals of the adsorbate’s interacting functional group can be used as a good metric for predicting the sign of the enantioselective binding, i.e., the identity of the preferred enantiomer. Collectively, these findings imply that during biorecognition the electron spin constraints contribute strongly to the enantiospecificity and necessitates a paradigm shift in how one should view selectivity in biorecognition.

## Supporting Information Available

General experiment methods, stability experiment of NAC assemblies, determination of adsorption and desorption behavior of LeuME on NAC, model for derivation of kinetic information, adsorption and desorption of l- and d-LeuME on different SAM, adsorption rate asymmetry of l-amino acids on l-NAC, control experiments on gold, absorbance and CD spectra of different l-amino acids and NAC. (PDF)
