# Supplementary material for: Beyond Stereoisomeric Effects: Exploring the Importance of Intermolecular Electron Spin Interactions in Biorecognition
Source: J Phys Chem Lett. 2023 Jul 31;14(31):7032–7. doi: 10.1021/acs.jpclett.3c01595 (PMC10424231; doi:10.1021/acs.jpclett.3c01595)
Supplement: Supplementary file 1 — jz3c01595_si_002.pdf [file jz3c01595_si_002.pdf]

Supporting information for

Beyond Stereoisomeric Effects: Exploring the Importance of Intermolecular Electron Spin Interactions in Biorecognition

Yiyang Lu[1], Meera Joy[1], Brian P. Bloom[1], David H. Waldeck[1*]

[1]Chemistry Department, University of Pittsburgh, Pittsburgh, Pennsylvania 15260, United States

Contents

Section 1 – Experimental method....S3

Magnetic electrochemical quartz crystal microbalance (mEQCM) measurements. S3

Self-assembled Monolayer Formation and Stability measurement. S3

Adsorption study with mEQCM. S3

Optical Measurements. S3

Section 2 - Stability of NAC assemblies S4

Section 3 Determination of Adsorption and Desorption Behavior of LeuME on NAC....S4

Section 4 Chronoamperometry Measurements and Data Analysis S6

Section 5 Adsorption Rate Asymmetry of L-amino acids on L-NAC....S12

Section 6 Control Experiments on Gold....S13

Section 7 Spectroscopic data S13

Figures

Fig. S1 (A) Linear sweep voltammograms and (B) corresponding mass changes of L-NAC (Orange) and D-NAC (Green) coated Ni/Au film in 0.1 M phosphate buffer. The Black arrow denotes the sweep direction. 4

Fig. S2 Panel (A) shows linear sweep voltammograms, and panel (B) the corresponding mass change, of an L-NAC coated Ni/Au film in 0.1 M phosphate buffer with 150 µM L-LeuME with a North (red) and South (blue) applied magnetic field. Panel (C) shows a zoomed in view of the mass change of (B). Panel (D) shows linear sweep voltammograms, and panel (E) shows the corresponding mass change, of an L-NAC coated Ni/Au film in 0.1 M phosphate buffer with 150 µM D-LeuME. Panel (F) shows a zoomed in view of the mass change of (E). The black arrow denotes the voltage sweep direction. 5

Fig. S3 Panel (A) shows linear sweep voltammograms, and panel (B) shows the corresponding mass change, of a D-NAC coated Ni/Au film in 0.1 M phosphate buffer with 150 µM L-LeuME, with a North (red) and a South (blue) applied magnetic field. Panel (C) shows a zoomed in view of the mass change in panel (B). Panel (D) shows linear sweep voltammograms, and panel (E) shows the corresponding mass change, of D-NAC coated Ni/Au film in 0.1 M phosphate buffer with 150 µM D-LeuME. Panel (F) shows a zoomed in view of the mass change in Panel (E). The black arrows denote the voltage sweep direction. 6

Fig. S4 (A) Chronoamperometry data for a 150 µM solution of L-LeuME. The applied potential sequence (top) and the corresponding frequency response (bottom) are recorded. (B) An expanded plot of the adsorption (top) and desorption (bottom) fits by an exponential decay or growth (red line) for determining the rate constant. (C) The histograms on the right comprise >150 fits of the adsorption (top) and desorption

(bottom) processes under a north magnetic field (red) and a south magnetic field (blue). A best fit of the data using a Gaussian distribution is shown as a solid curve. 7
Fig. S5 The average mass change during adsorption of L-LeuME onto Ni/Au film coated with (A) L-NAC, (B) MPA, and (C) D-NAC SAMs under North magnetic field (red) and South magnetic field (blue). The average mass change during adsorption of D-LeuME onto Ni/Au film coated with (D) L-NAC, (E) MPA, (F) and D-NAC SAMs under North magnetic field (red) and South magnetic field (blue). 8
Fig. S6 Histograms of adsorption rate constant for D-LeuME onto Ni/Au films coated with (A) L-NAC, (B) MPA, and (C) D-NAC SAMs under North magnetic field (red) and South magnetic field (blue). Panel (D) shows the average mass change during the adsorption of D-LeuME on to Ni/Au film coated with different SAMs under a North magnetic field (red) and a South magnetic field (blue). 9
Fig. S7 Histograms of desorption rate constants of L-LeuME from Ni/Au film coated with (A) L-NAC, (B) MPA, and (C) D-NAC SAM, and the average mass change during desorption of L-LeuME from Ni/Au films coated with (D) L-NAC, (E) MPA, and (F) D-NAC SAMs under North magnetic field (red) and South magnetic field (blue). 10
Fig. S8 Histograms for the desorption rate constants of D-LeuME from Ni/Au film coated with (A) L-NAC, (B) MPA, and (C) D-NAC SAMs, and the average mass change during desorption of D-LeuME from Ni/Au film coated with (D) L-NAC, (E) MPA, and (F) D-NAC SAMs under North magnetic field (red) and South magnetic field (blue). 11
Fig. S9 Panel (A) shows the polarization in desorption rate constant of LeuME enantiomers on different SAMs. Panel (B) shows the polarization in adsorption rate constant of Phe enantiomers on different SAM. 11
Fig. S10 Histograms of adsorption rate constants of 150 μM (A) L-Phe, (B) L-Trp, (C) L-His, (D) L-Leu, (E) L-Ile, (F) L-Pro, (G) L-Ser, (H) L-Ala, (I) L-Tyr, (J) L-LeuME, (K) Gly onto L-NAC SAM coated Ni/Au films under North magnetic field (red) and South magnetic field (blue). A best fit of the data using a Gaussian distribution is shown as a solid line...12
Fig. S11 Histograms for the adsorption rate constant of 150 μM (A) L-LeuME and (B) D-LeuME and (C) L-Phe onto L-NAC SAM coated Au films under North magnetic field (red) and South magnetic field (blue). A best fit of the data using a Gaussian distribution is shown as a solid line. 13
Fig. S12 Absorbance and CD spectra of 1.5 mM L-NAC (Orange) and D-NAC (Green) in 2 mM phosphate buffer solutions at pH 9..13
Fig. S13 Absorbance spectra of of 0.5 mM solutions of the different amino acids studied in 2 mM phosphate buffer solution at pH 9; (A) L-Phe, (B) L-Trp, (C) L-His, (D) L-Leu, (E) L-Ile, (F) L-Ser, (G) L-Ala, (H) L-Pro, (I) L-Tyr, (J) L-LeuME, (K) Gly. 14
Fig. S14 CD spectra of 0.5 mM solutions of the different amino acids studied in 2 mM phosphate buffer solution at pH 9; (A) L-Phe, (B) L-Trp, (C) L-His, (D) L-Leu, (E) L-Ile, (F) L-Ser, (G) L-Ala, (H) L-Pro, (I) L-Tyr, (J) L-LeuME, (K) Gly. 15

Section 1 – Experimental method

Magnetic electrochemical quartz crystal microbalance (mEQCM) measurements.

The mEQCM experiments were performed using a 7.9995 MHz quartz crystal in an EQCM cell attachment and a 430A potentiostat (CH Instruments). The surface area of the crystal is 0.205 cm^2 and is coated with 100 nm of nickel and 10 nm polycrystalline gold as the working electrode area (CH Instruments). The counter electrode was a Pt wire and the reference electrode was a saturated Ag/AgCl electrode. Magnetic field studies placed a permanent magnet, 0.54 T (K&J Magnetics) underneath the working electrode during the experiment.

Self-assembled Monolayer Formation and Stability measurement.

The quartz crystal working electrodes were incubated in a 50 mM solution of L-NAC (or D-NAC) in 0.1 M phosphate buffer at pH 9 overnight. Then the electrode was rinsed three times with phosphate buffer and water to remove any loosely, physisorbed material. The electrodes were then blown dry with a stream of Argon. Linear sweep voltammetry (LSV) and QCM measurements were used to study the stability of the L- and D-NAC SAMs. Here, the SAM coated quartz crystal working electrode was incubated in a 0.1 M phosphate buffer (pH 9) electrolyte solution for 30 min, to allow the QCM frequency to stabilize. Next the electrode was scanned from 0 V to – 0.8 V versus saturated Ag|AgCl at a scan rate = 25 mV/s and the change in mass was monitored.

Adsorption study with mEQCM.

The SAM coated quartz crystal was first incubated in a 0.1 M phosphate buffer (pH 9) electrolyte solution containing a 150 μM of an amino acid and allowed to equilibrate for 30 minutes. Next, linear sweep voltammetry and QCM measurements were performed to determine the desorption and re-adsorption of the amino acid onto the NAC SAM coated film; the data were collected by scanning from -0 V to – 0.8 V versus saturated Ag|AgCl at a scan rate = 25 mV/s. Upon determination of the potentials required for adsorption and desorption, the SAM coated quartz crystal was incubated for an additional 30 min to allow the system to equilibrate and then chronoamperometry experiments were performed. Take for example L-LeuME; An initial potential of -0.4 V corresponding to reductive adsorption of L-LeuME was applied and then a more positive potential, 0 V, corresponding to oxidative desorption. The applied voltage was jumped between these two values and the QCM response was collected as a function of time after the potential jump. A 5 s pulse width was applied during the experiment and >150 cycles were performed to calculate the average mass change for thermodynamic analysis and to build up the statistics for histogram plots to perform a kinetic analysis of the adsorption and desorption process. It is important to stress that the measurements made using North and South magnetizations occur on the same NAC film and therefore any imperfections in the quality of the SAM should not influence the observed spin effects.

Optical Measurements.

Absorbance spectroscopy was performed using an Agilent model 8453 spectrometer and Circular dichroism (CD) spectra were measured using a JASCO J-810 CD spectrometer with a scan rate of 20 nm/min and a bandwidth of 1 nm. For the CD measurements each sample was scanned three times and the average of the three curves is reported. The absorbance and circular dichroism data were collected in a 2 mm quartz cuvette containing 2 mM phosphate buffer (pH 9) and 0.5mM concentration of amino acid.

Section 2 - Stability of NAC assemblies

Linear sweep voltammetry measurements were performed to determine the potential required for desorption and re-adsorption of LeuME enantiomers from the NAC coated Ni/Au films. First, the stability of L-NAC and D-NAC coated Ni/Au film was studied. When scanning negatively from 0 V to -0.8V, a cathodic desorption peak occurs at around -0.65 V for both L-NAC and D-NAC (Figure S1A), and the mass begins to decrease from -0.45 V (Figure S1B). These results indicate that the L-NAC and D-NAC are stable if the applied potential is more positive than -0.4 V.

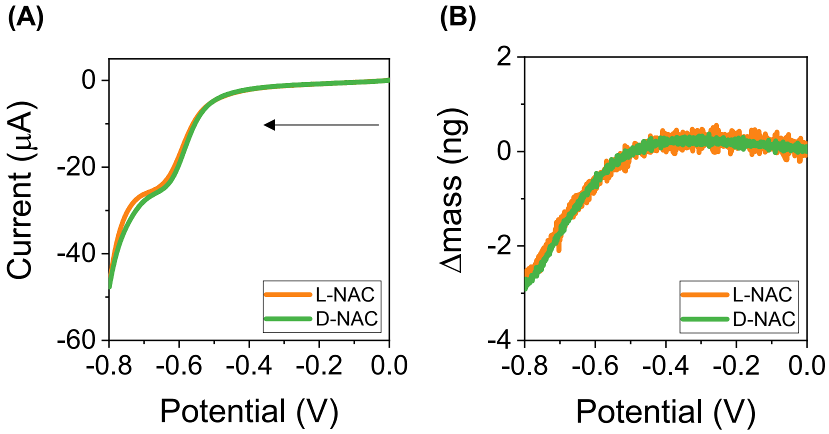

Fig. S1 (A) Linear sweep voltammograms and (B) corresponding mass changes of L-NAC (Orange) and D-NAC (Green) coated Ni/Au film in 0.1 M phosphate buffer. The Black arrow denotes the sweep direction.

Section 3 Determination of Adsorption and Desorption Behavior of LeuME on NAC

To determine the adsorption of LeuME enantiomers onto NAC coated Ni/Au films the potential was scanned from 0 V to -0.8V and the change in mass studied. As the potential was swept, a mass increase process followed by a mass decrease process was observed (Figure S2B & E). This behavior is attributed to cathodic adsorption of LeuME enantiomers onto the L-NAC SAM surface (0 V to around -0.4V) that is followed by a complete cathodic desorption of L-NAC with LeuME (-0.4V to -0.8V). Interestingly, more L-LeuME adsorbs onto the surface under a North magnetic field (red) than a South magnetic field (blue); See Figure S2 C. Conversely the opposite behavior manifests for D-LeuME (Figure S2F). Also, the desorption peak of L-NAC with LeuME exhibits a dependence on the enantiomeric form of LeuME and the magnetic field direction; L-LeuME on L-NAC shows a more negative desorption peak under a North magnetic field, indicating a stronger binding onto Ni/Au film, whereas D-LeuME on L-NAC shows the opposite dependence on magnetic field. These results indicate that the charge delocalization across the NAC-LeuME composite is spin-dependent and enantiospecific. Fig. S3 shows complimentary measurements on D-NAC SAMs, and the result is opposite to that on L-NAC SAMs.

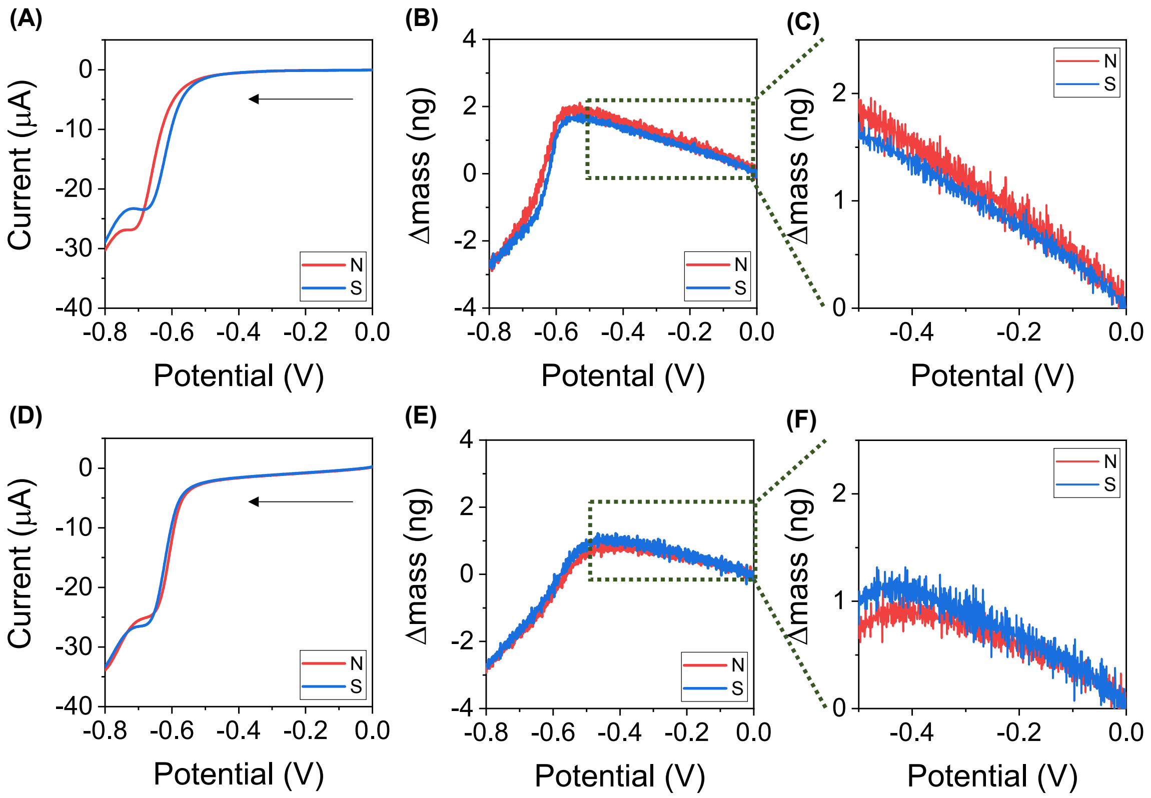

Fig. S2 Panel (A) shows linear sweep voltammograms, and panel (B) the corresponding mass change, of an L-NAC coated Ni/Au film in 0.1 M phosphate buffer with 150 μM L-LeuME with a North (red) and South (blue) applied magnetic field. Panel (C) shows a zoomed in view of the mass change of (B). Panel (D) shows linear sweep voltammograms, and panel (E) shows the corresponding mass change, of an L-NAC coated Ni/Au film in 0.1 M phosphate buffer with 150 μM D-LeuME. Panel (F) shows a zoomed in view of the mass change of (E). The black arrow denotes the voltage sweep direction.

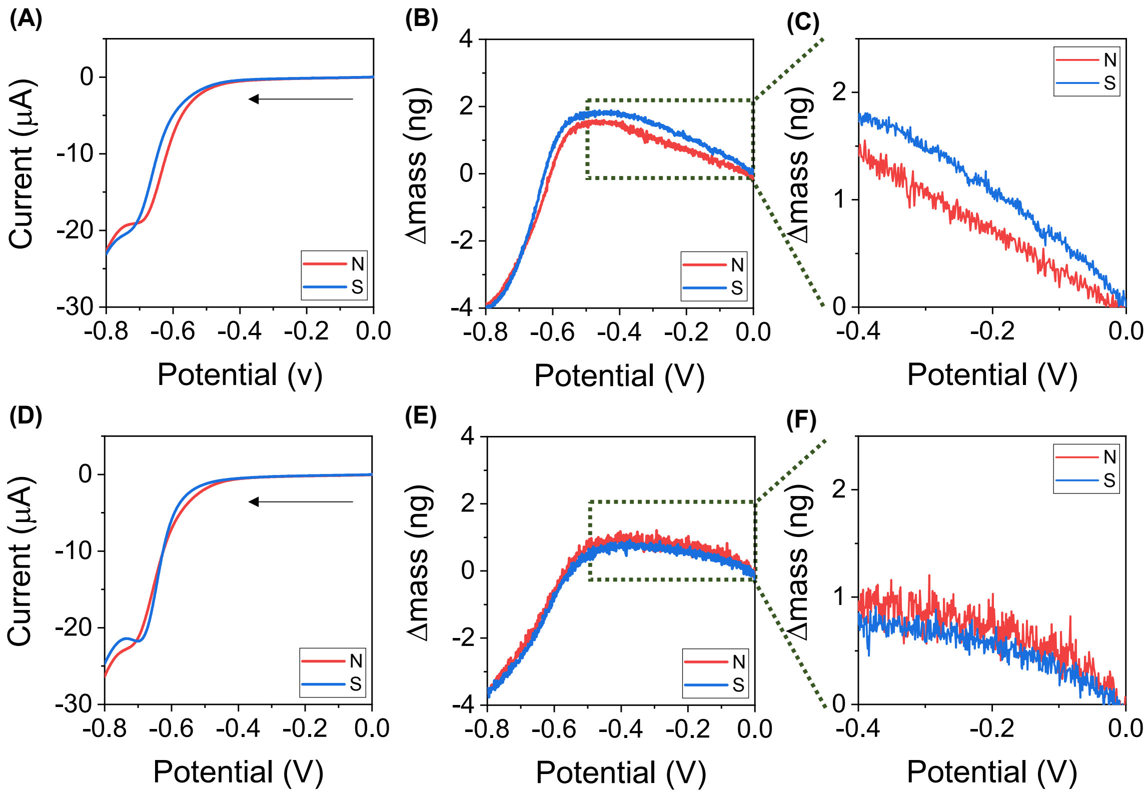

Fig. S3 Panel (A) shows linear sweep voltammograms, and panel (B) shows the corresponding mass change, of a D-NAC coated Ni/Au film in 0.1 M phosphate buffer with 150 μM L-LeuME, with a North (red) and a South (blue) applied magnetic field. Panel (C) shows a zoomed in view of the mass change in panel (B). Panel (D) shows linear sweep voltammograms, and panel (E) shows the corresponding mass change, of D-NAC coated Ni/Au film in 0.1 M phosphate buffer with 150 μM D-LeuME. Panel (F) shows a zoomed in view of the mass change in Panel (E). The black arrows denote the voltage sweep direction.

Section 4 Chronoamperometry Measurements and Data Analysis

Figure S4A shows representative data for the chronoamperometry measurements; the top panel shows the potential sequence and the bottom panel shows the corresponding frequency response. To extrapolate the rate constant data from the frequency response, the following calculations were performed. First, the adsorption process was assumed to follow a simple Langmuir model and thus,the adsorption rate can be written as Eq. (1)

$$r_{ads} = \frac{d\theta}{dt} = k_{ads} \cdot c_{Leu\,ME} \cdot (1-\theta) = k'_{ads} \cdot (1-\theta) \qquad \text{Eq. (1)}$$

where $c_{Leu\,ME}$ is the concentration of LeuME in solution, θ is the concentration of adsorbed molecule on the surface, k_{ads} is the adsorption rate constant and k'_{ads} is the effective adsorption rate constant. To quantify the adsorption rates, the time responses of the QCM frequency were fit to an exponential decay equation $y = A \cdot e^{-t/\tau_1} + y_0$ for adsorption (Figure S4B top panel), where A, y_0, and τ_1 were adjusted for a best fit to the data. Then the effective adsorption rate constant

was calculated as $k'_{ads} = \frac{1}{|\tau_1|}$. A histogram with >150 k'_{ads}, determined through this method was then built and fit to a Gaussian distribution to obtain the average and standard deviation of the mean for the adsorption rate constant (Figure S4C top panel).

The desorption rate can be written as Eq. (2)

$$r_{des} = -\frac{d\theta}{dt} = k_{des} \cdot \theta \qquad \text{Eq. (2)}$$

where θ is the concentration of adsorbed molecule on the surface and k_{des} is the desorption rate constant. The time responses of the QCM frequency were fit to an exponential growth equation $y = A \cdot e^{t/\tau_2} + y_0$ for desorption (Fig, S4B bottom panel), where A, y_0, and τ_2 were adjusted for a best fit to the data. Then, the desorption rate constant was calculated as $k_{des} = \frac{1}{|\tau_2|}$. A histogram with >150 k_{des}, determined through this method was then built and again fit to a Gaussian distribution to obtain the average and standard deviation of the mean for the desorption rate constant (Figure S4C bottom panel).

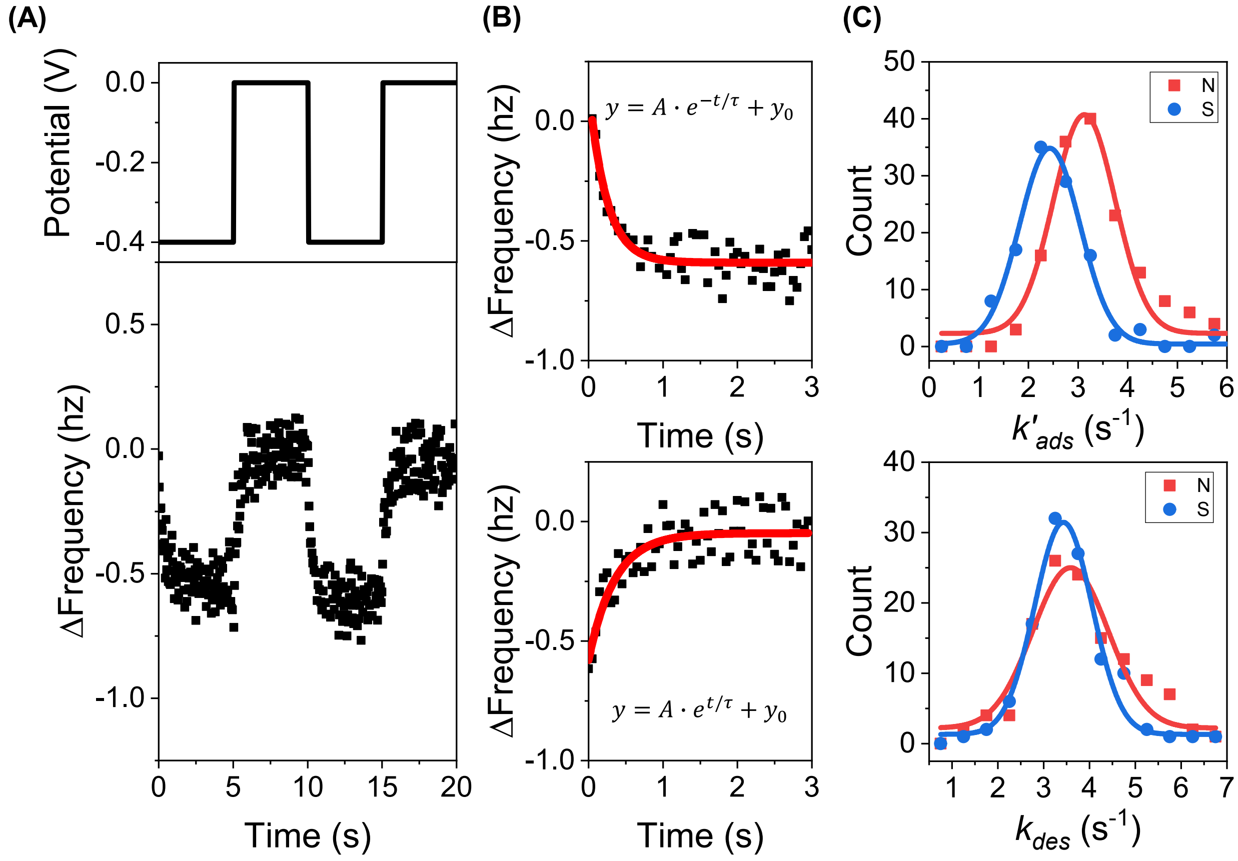

Fig. S4 (A) Chronoamperometry data for a 150 μM solution of L-LeuME. The applied potential sequence (top) and the corresponding frequency response (bottom) are recorded. (B) An expanded plot of the adsorption (top) and desorption (bottom) fits by an exponential decay or growth (red line) for determining the rate constant. (C) The histograms on the right comprise >150 fits of the adsorption (top) and desorption

(bottom) processes under a north magnetic field (red) and a south magnetic field (blue). A best fit of the data using a Gaussian distribution is shown as a solid curve.

To quantify the mass change during the adsorption (desorption) process, eighty QCM frequency responses were selected at random and the average change in frequency during the 5s adsorption (desorption) process was quantified. A frequency shift of -1.0 Hz corresponds to a mass change of 1.4 ng based on the Sauerbrey equation and characteristics of our EQCM set-up. To determine the mass change, the data were fit to an exponential growth equation, $y = A \cdot e^{t/\tau_1} + y_0$ (Fig. S4), in which A, y_0, and τ_1 and were adjusted for a best fit to the data, allowing the coverage after 5s to be calculated. Fig. S5 (A) –(C) replots the average mass change, reported in Fig. 3 (A)-(C) in the main text, for L-LeuME adsorption on L-NAC, MPA, and D-NAC, and the complimentary measurements on the same substrates for D-LeuME are plotted in Figure S5(D)-(F).

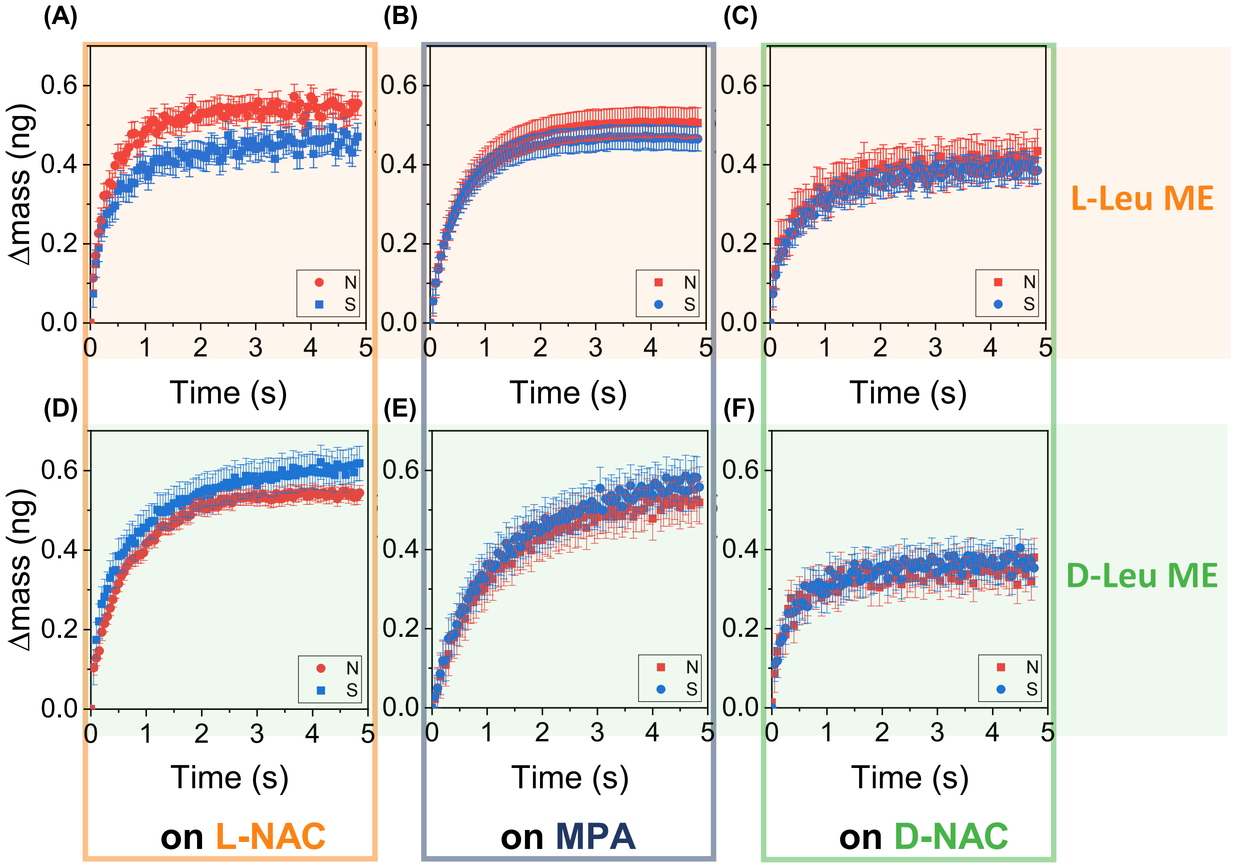

Fig. S5 The average mass change during adsorption of L-LeuME onto Ni/Au film coated with (A) L-NAC, (B) MPA, and (C) D-NAC SAMs under North magnetic field (red) and South magnetic field (blue). The average mass change during adsorption of D-LeuME onto Ni/Au film coated with (D) L-NAC, (E) MPA, (F) and D-NAC SAMs under North magnetic field (red) and South magnetic field (blue).

Figure S6 shows histograms for the adsorption rate constants of D-LeuME onto L-NAC (Figure S6A), MPA (Figure S6B), and D-NAC (Figure S6C) coated electrodes upon application of a North

(red) and South (blue) magnetic field. In all three SAM configurations the adsorption rate constant for D-LeuME is faster when the magnetic field is oriented South rather than North. The change in mass of D-LeuME at 5s, after the kinetically controlled adsorption process is shown in Figure S6D. Values for the mass change in Figure S6D and Figure 3 in the main text are reported in Table S1. Homochiral ensembles exhibited the largest average mass (L-LeuME on L-NAC, and D-LeuME on D-NAC). Heterochiral ensembles exhibited the smallest average mass (L-LeuME on D-NAC, and D-LeuME on L-NAC). Achiral SAMs were intermediate. Analogous adsorption rate constant measurements and mass changes occurring during the desorption process are shown in Figure S7 and S8. for L-LeuME and D-LeuME, respectively, on L-NAC, MPA, and D-NAC SAMs. A summary of the polarization in adsorption rate constant for LeuME desorption is shown in Figure S9A and complimentary measurements on phenylalanine, Phe, for adsorption is shown in Figure S9B.

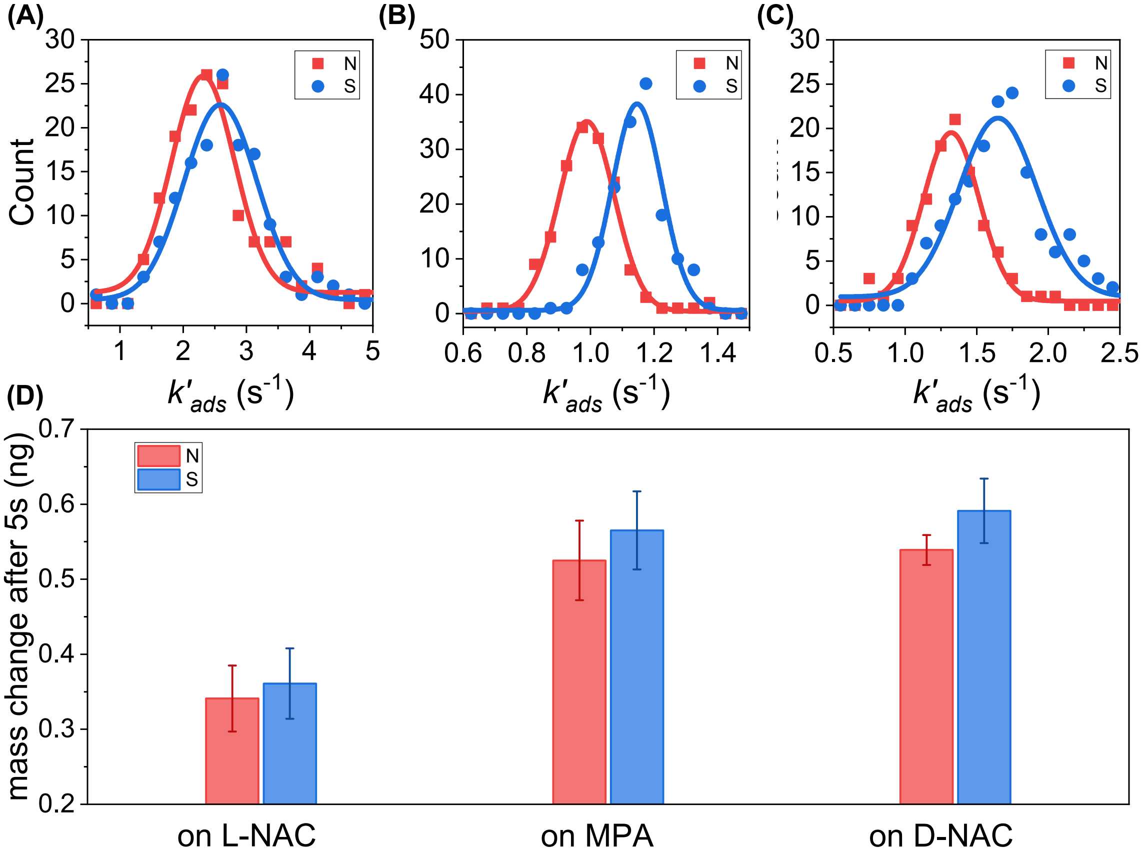

Fig. S6 Histograms of adsorption rate constant for D-LeuME onto Ni/Au films coated with (A) L-NAC, (B) MPA, and (C) D-NAC SAMs under North magnetic field (red) and South magnetic field (blue). Panel (D) shows the average mass change during the adsorption of D-LeuME on to Ni/Au film coated with different SAMs under a North magnetic field (red) and a South magnetic field (blue).

Table S1. Total mass change (in unit of ng) for the adsorption of L-LeuME and D-LeuME onto different SAMs after 5s

	L-LeuME			D-LeuME		
	N	S	average	N	S	average
L-NAC	0.566±0.034	0.447±0.030	0.506±0.032	0.341±0.024	0.361±0.027	0.351±0.026
MPA	0.503±0.038	0.466±0.030	0.485±0.034	0.525±0.023	0.565±0.022	0.545±0.023
D-NAC	0.404±0.054	0.381±0.032	0.393±0.043	0.539±0.010	0.591±0.023	0.565±0.016

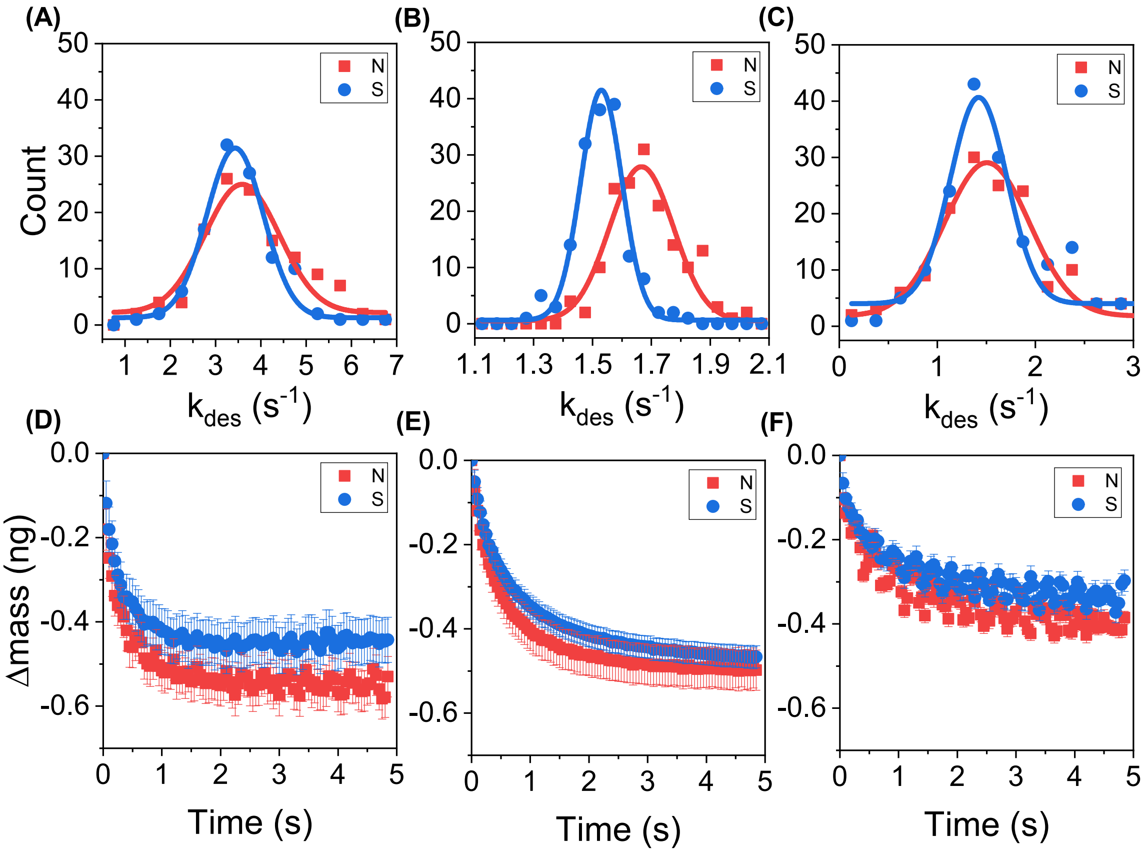

Fig. S7 Histograms of desorption rate constants of L-LeuME from Ni/Au film coated with (A) L-NAC, (B) MPA, and (C) D-NAC SAM, and the average mass change during desorption of L-LeuME from Ni/Au films coated with (D) L-NAC, (E) MPA, and (F) D-NAC SAMs under North magnetic field (red) and South magnetic field (blue).

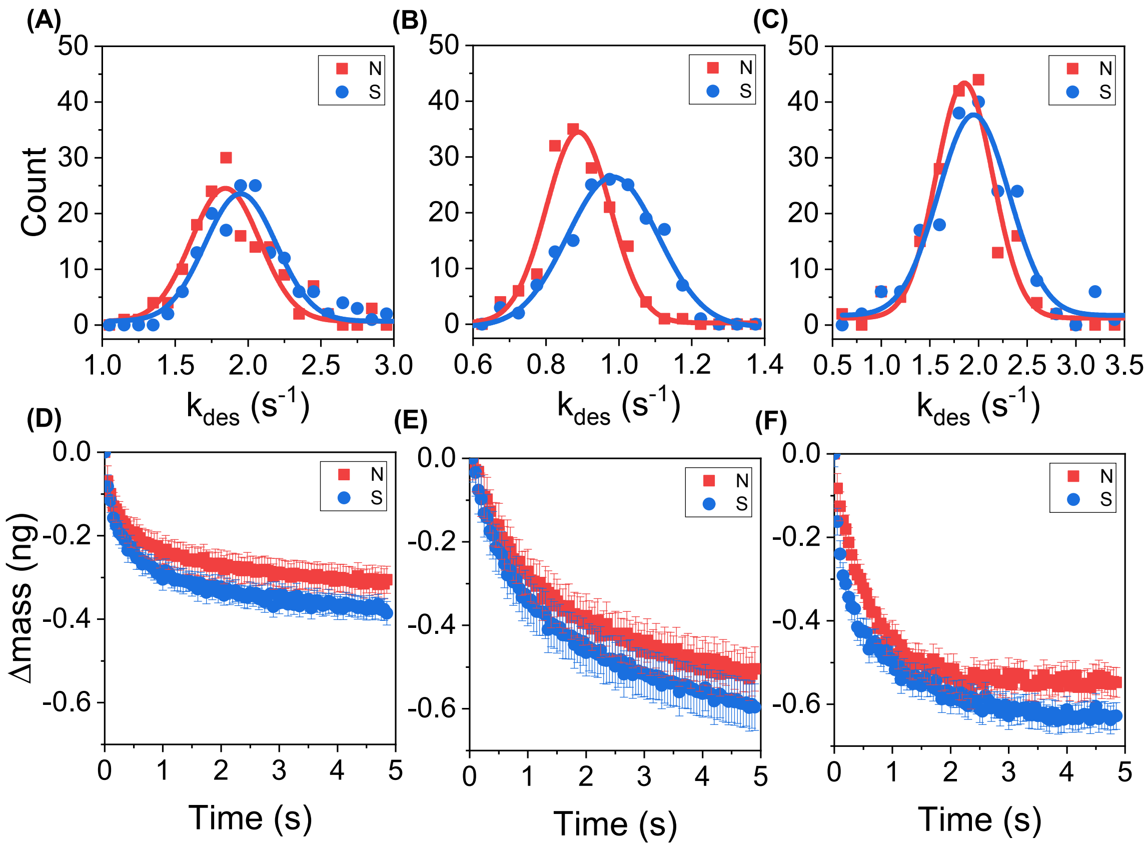

Fig. S8 Histograms for the desorption rate constants of D-LeuME from Ni/Au film coated with (A) L-NAC, (B) MPA, and (C) D-NAC SAMs, and the average mass change during desorption of D-LeuME from Ni/Au film coated with (D) L-NAC, (E) MPA, and (F) D-NAC SAMs under North magnetic field (red) and South magnetic field (blue).

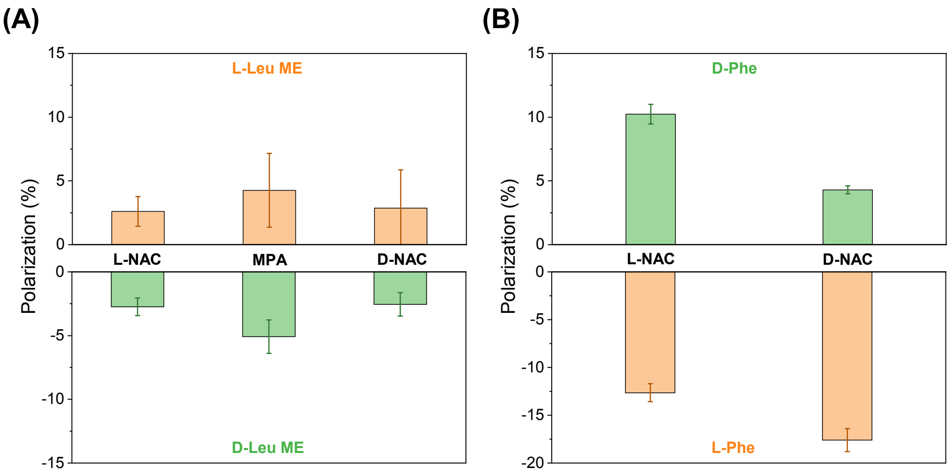

Fig. S9 Panel (A) shows the polarization in desorption rate constant of LeuME enantiomers on different SAMs. Panel (B) shows the polarization in adsorption rate constant of Phe enantiomers on different SAM.

Section 5 Adsorption Rate Asymmetry of L-amino acids on L-NAC

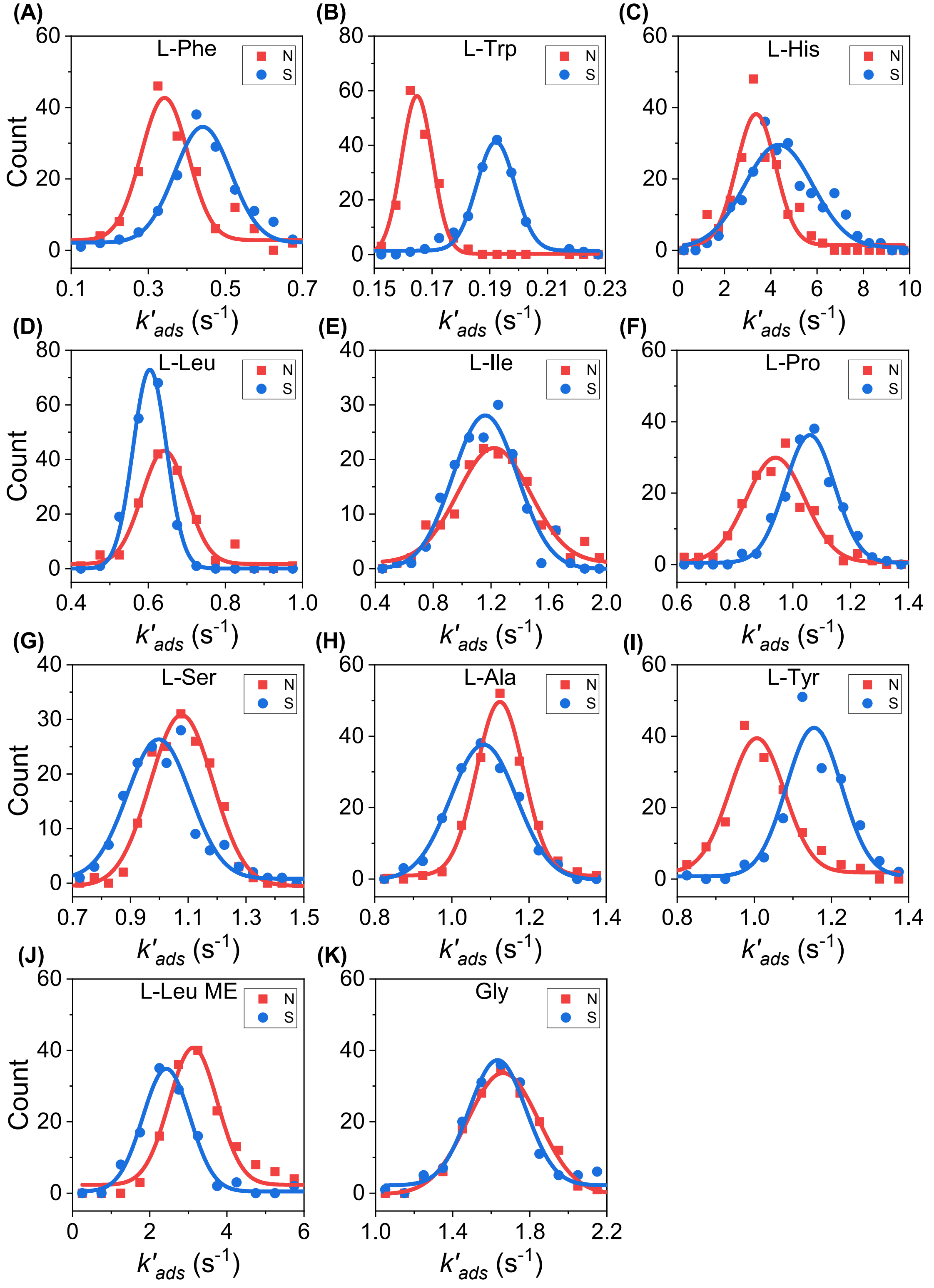

Fig. S10 Histograms of adsorption rate constants of 150 μM (A) L-Phe, (B) L-Trp, (C) L-His, (D) L-Leu, (E) L-Ile, (F) L-Pro, (G) L-Ser, (H) L-Ala, (I) L-Tyr, (J) L-LeuME, (K) Gly onto L-NAC SAM coated

Ni/Au films under North magnetic field (red) and South magnetic field (blue). A best fit of the data using a Gaussian distribution is shown as a solid line.

Section 6 Control Experiments on Gold

Control experiments were performed for the adsorption of L-LeuME and L-Phe onto L-NAC SAM coated Au films, which are not ferromagnetic, under North and South magnetic fields; See Fig. S11. The polarization in adsorption rate constant for L-LeuME is 0.94 ± 0.64% and for L-Phe is 1.09 ± 0.58%, indicating the adsorption behavior of a chiral molecule onto a nonmagnetic substrate is the same under the North and South magnetic field. Thus, the enantiospecific adsorption is not arising from the magnetic field per se, but instead from the CISS-mediated spin dependent exchange interactions at the ferromagnetic surface.

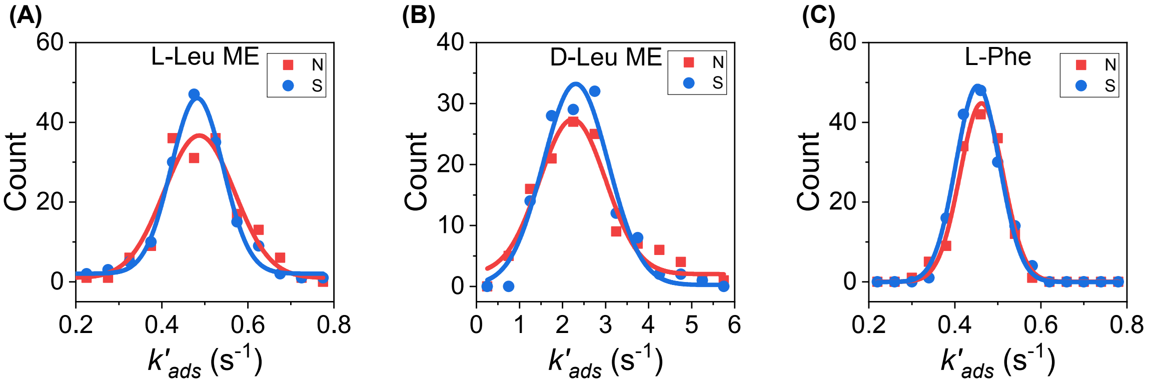

Fig. S11 Histograms for the adsorption rate constant of 150 μM (A) L-LeuME and (B) D-LeuME and (C) L-Phe onto L-NAC SAM coated Au films under North magnetic field (red) and South magnetic field (blue). A best fit of the data using a Gaussian distribution is shown as a solid line.

Section 7 Spectroscopic data

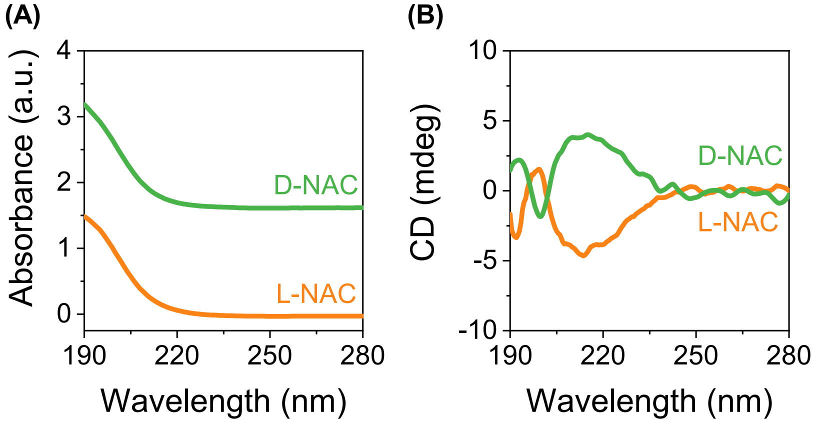

Fig. S12 Absorbance and CD spectra of 1.5 mM L-NAC (Orange) and D-NAC (Green) in 2 mM phosphate buffer solutions at pH 9.

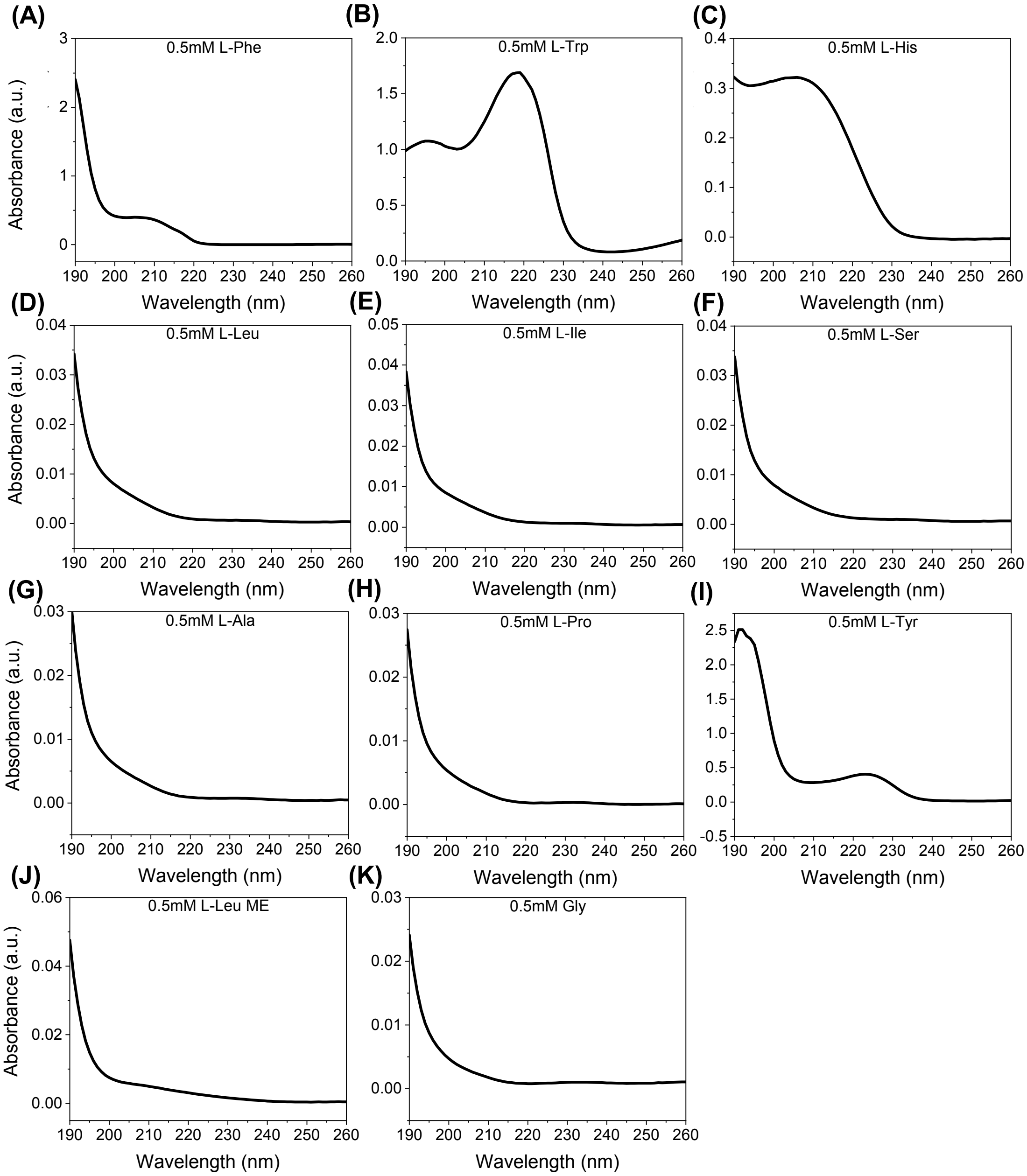

Fig. S13 Absorbance spectra of of 0.5 mM solutions of the different amino acids studied in 2 mM phosphate buffer solution at pH 9; (A) L-Phe, (B) L-Trp, (C) L-His, (D) L-Leu, (E) L-Ile, (F) L-Ser, (G) L-Ala, (H) L-Pro, (I) L-Tyr, (J) L-LeuME, (K) Gly.

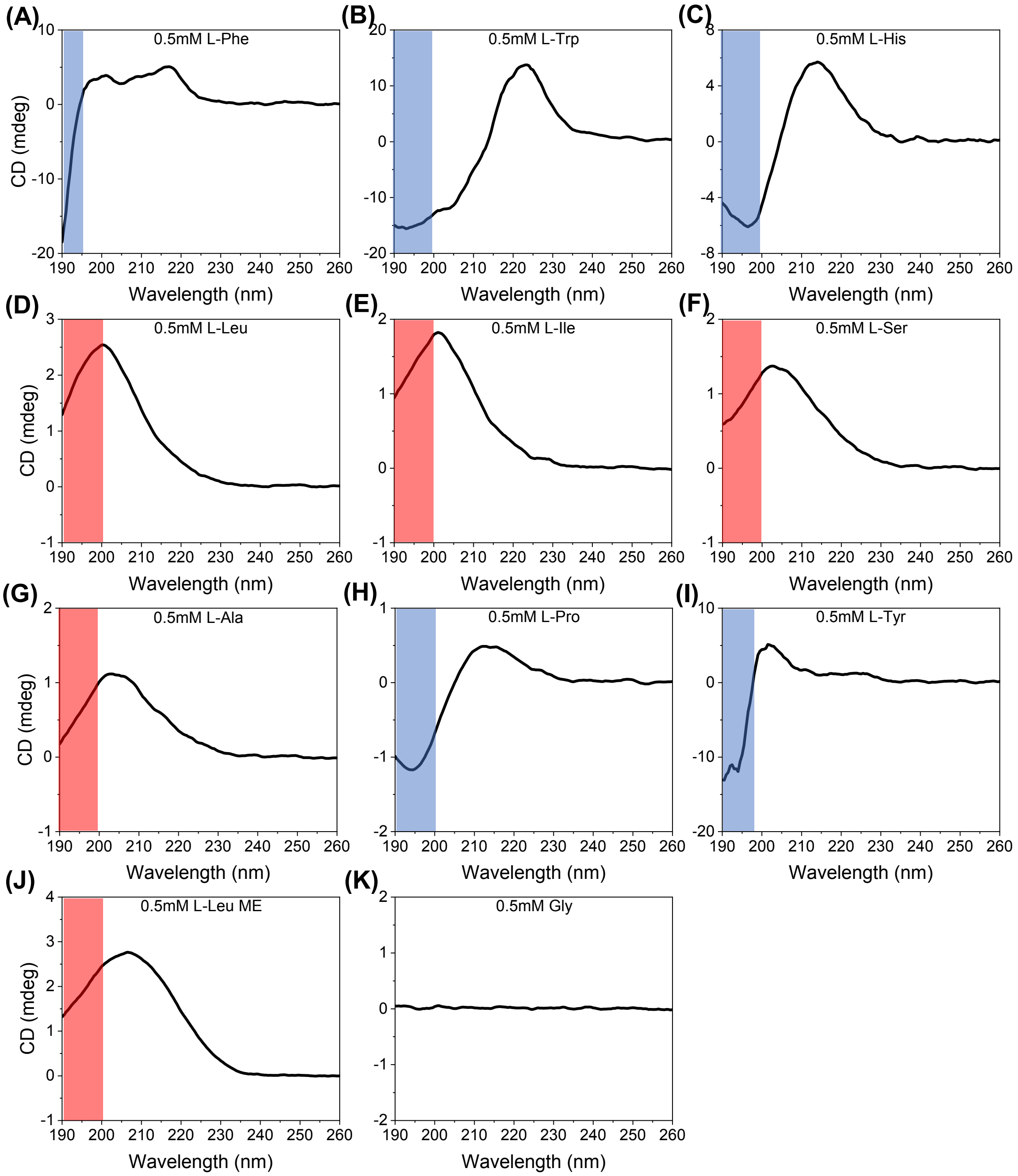

Fig. S14 CD spectra of 0.5 mM solutions of the different amino acids studied in 2 mM phosphate buffer solution at pH 9; (A) L-Phe, (B) L-Trp, (C) L-His, (D) L-Leu, (E) L-Ile, (F) L-Ser, (G) L-Ala, (H) L-Pro, (I) L-Tyr, (J) L-LeuME, (K) Gly.
